# An untargeted multi-technique metabolomics approach to studying intracellular metabolites of HepG2 cells exposed to 2,3,7,8-tetrachlorodibenzo-p-dioxin

**DOI:** 10.1186/1471-2164-12-251

**Published:** 2011-05-20

**Authors:** Ainhoa Ruiz-Aracama, Ad Peijnenburg, Jos Kleinjans, Danyel Jennen, Joost van Delft, Caroline Hellfrisch, Arjen Lommen

**Affiliations:** 1 RIKILT-Institute of Food Safety, Wageningen University and Research Centre, P.O. Box 230, 6700 AE, Wageningen, The Netherlands; 2 Department of Health Risk Analysis and Toxicology, P.O. Box 616, 6200 MD Maastricht University, Maastricht, The Netherlands; 3 Netherlands Toxicogenomics Centre, The Netherlands

## Abstract

**Background:**

*In vitro *cell systems together with omics methods represent promising alternatives to conventional animal models for toxicity testing. Transcriptomic and proteomic approaches have been widely applied *in vitro *but relatively few studies have used metabolomics. Therefore, the goal of the present study was to develop an untargeted methodology for performing reproducible metabolomics on *in vitro *systems. The human liver cell line HepG2, and the well-known hepatotoxic and non-genotoxic carcinogen 2,3,7,8-tetrachlorodibenzo-p-dioxin (TCDD), were used as the *in vitro *model system and model toxicant, respectively.

**Results:**

The study focused on the analysis of intracellular metabolites using NMR, LC-MS and GC-MS, with emphasis on the reproducibility and repeatability of the data. State of the art pre-processing and alignment tools and multivariate statistics were used to detect significantly altered levels of metabolites after exposing HepG2 cells to TCDD. Several metabolites identified using databases, literature and LC-nanomate-Orbitrap analysis were affected by the treatment. The observed changes in metabolite levels are discussed in relation to the reported effects of TCDD.

**Conclusions:**

Untargeted profiling of the polar and apolar metabolites of *in vitro *cultured HepG2 cells is a valid approach to studying the effects of TCDD on the cell metabolome. The approach described in this research demonstrates that highly reproducible experiments and correct normalization of the datasets are essential for obtaining reliable results. The effects of TCDD on HepG2 cells reported herein are in agreement with previous studies and serve to validate the procedures used in the present work.

## Background

Metabolomics has been defined as the quantitative measurement of the multi-parametric metabolic response of living systems to patho-physiological stimuli or genetic modification [1]. It encompasses the qualitative and quantitative measurement of metabolites interacting in a biological system; targeted and untargeted strategies for analysis of metabolites can be used. Targeted studies focus on the analysis of a predefined list of metabolites, whereas the initial objective of untargeted metabolomics is to analyze as many non-predefined metabolites as possible at the raw signal level. With the latter approach, identification is only carried out on relevant signals [2,3].

Recently, there has been an exponential growth in the number of published papers concerning metabolomics of a wide variety of systems [4-7]. Metabolomic approaches have been used for toxicological studies [8]. However, in most cases, biofluids or tissues from *in vivo *experiments have been analyzed [8,9]. Few toxicological studies have been published that concern the profiling of intracellular metabolites using *in vitro *cell culture systems [10,11]

Owing to ethical concerns (animal welfare) and cost efficiency, there is a need to develop alternatives to conventional toxicity testing incorporating animals [12]. Among these alternatives, *in vitro *systems are considered particularly promising [13,14]. Much research has focused on the analysis of the effects of toxic compounds using *in vitro *systems and omics techniques [15-18]. However, transcriptomics and proteomics have predominantly been used to elucidate the toxic mechanisms of the studied compounds.

The goals of the present work were two-fold. First, to develop an untargeted *in vitro *cell system methodology with reproducible metabolomics; second, to evaluate toxicant-induced cell responses on metabolic levels with regards to published data concerning the toxicant, thereby substantiating the methodology. TCDD (2,3,7,8-tetrabenzodi-p-dioxin) was chosen as the model toxic compound as it has been widely studied *in vivo *and *in vitro *[15,19,20], particularly in terms of its hepatotoxic, carcinogenic and immunotoxic effects. Toxic effects of dioxins mediated by the aryl hydrocarbon receptor (AhR) include the wasting syndrome [21], the induction of oxidative damage [22,23], hepatic injury and carcinogenesis [24,25]. TCDD has also been reported to have an anti-proliferative effect [26].

TCDD is an agonist of AhR, a cytosolic ligand-activated transcription factor. Upon activation, AhR dimerizes with ARNT to form a heterodimer that binds to DNA sequences called xenobiotic response elements (XREs). Through such binding, AhR up-regulates the expression of several downstream genes including those encoding xenobiotic metabolizing enzymes such as Phase I (e.g. cytochrome P450 monooxygenases) and Phase II (e.g. glutathione S-transferases, sulfotransferases) biotransformation enzymes [27].

In this study, the human hepatoma cell line HepG2 was chosen for experiments concerning *in vitro *exposure to TCDD as this compound is a well known liver toxicant. HepG2 cells have preserved the activities of several phase I and phase II enzymes [28]. Consequently, HepG2 cells have been widely used as a model for various omics studies concerning carcinogenicity and hepatotoxicity [28-30]. Furthermore, this cell line has been exploited for studying the effects of TCDD on gene expression using transcriptomics [15-17,26].

For the untargeted metabolomics approach used in this study, multiple complementary analytical techniques were applied to polar and apolar cell lysate fractions, i.e. NMR and GCMS to the apolar fraction, and NMR and LCMS to the polar fraction. Data analysis was performed using state-of-the-art software [31-33] for pre-processing and alignment of data sets in combination with multivariate statistical analysis and advanced identification technology.

Particular emphasis was placed on the repeatability of experiments, reproducibility of metabolic changes, normalization and validation of the results by literature comparison.

## Results

The advantages and limitations of omics techniques applied to *in vitro *systems must be elucidated before *in vitro *omics-based alternatives to conventional toxicity studies are considered valid. The goal of the present study was to develop a reproducible untargeted metabolomics methodology for *in vitro *studies using the HepG2 human hepatocarcinoma cell line exposed to TCDD for 48 h.

Cell extracts containing intracellular metabolites were separated into polar and apolar fractions and subjected to 1D ^1^H-NMR and LC-MS or 1D ^1^H-NMR and GC-MS, respectively. The majority of NMR and MS data obtained were complementary in terms of their information content. Raw data were pre-processed and aligned using specific software enabling statistical analysis. Subsequently, metabolites with significantly altered levels were identified using databases, literature and LC-nanomate-Orbitrap analysis. In this study, a particular focus concerned the repeatability of experiments with HepG2 cells obtained from different passages, and reproducibility of biological replicates from the same passage, and normalization of experimental data prior to comparison. Results were compared with data from previous studies concerning the effects of TCDD in non-metabolomics studies.

The first major problem addressed was the normalization of chemical profiles. In this study, typical changes in metabolite concentrations were between 20 and 100%. Compounds such as TCDD can influence overall cell metabolism and cause a decrease in the rate of cell proliferation. Therefore, the net effect of exposure can be an overall decrease of metabolites because there is less cell material. If this is not taken into account it will dominate the metabolomics results and metabolic effects may go undetected. Furthermore, in order to identify statistically significant changes in metabolite concentrations in the order of 20 to 100%, it was essential to have multiple replicates.

Preliminary studies in our laboratory have shown that the age of cell cultures (expressed as passage number) could influence the magnitude of the effect of the exposure on the cell metabolome. To address this issue, HepG2 cells obtained from different passage numbers were exposed to medium containing 10 nM TCDD or the vehicle, DMSO, for a period of 48 h.

### The apolar fraction

**Figure 1:**
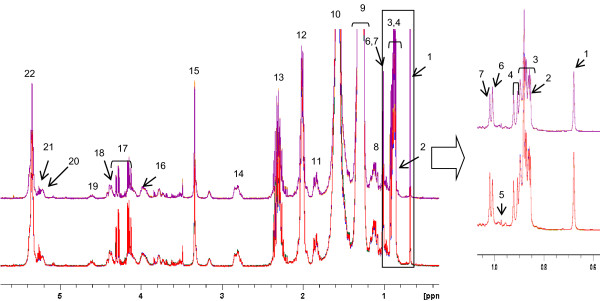
**Total **^**1**^**H NMR spectral signals of the apolar fraction of HepG2 cells exposed to TCDD (above) or the vehicle control DMSO (below)**. For both treatments, four spectra were overlaid. The numbers of the signals correspond to those indicated in Table 1.

**Figure 2:**
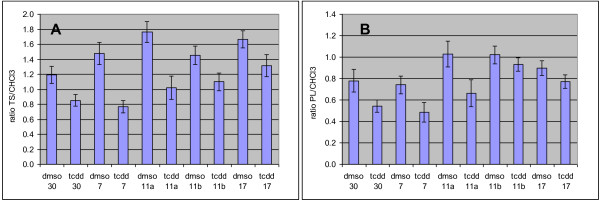
**Ratios between the intensity of some NMR spectral signals**. A: Ratio between the intensity of the total NMR spectral signals (TS) and the intensity of the residual CHCl_3 _signal. B: Ratio between the intensity of the phospholipid signal in the NMR spectra (PL) and the intensity of the residual CHCl_3 _signal.

NMR analyses of the apolar fraction were performed using the same deuterated chloroform. Therefore, the residual proton signal of chloroform was used as an initial absolute internal standard. This allowed the amount of lipids in solution to be estimated by calculating the ratio between the sum of the intensities of all spectral signals (TS (total signals) = signals 1-22 in Figure 1) and the intensity of the residual CHCl_3 _signal (not shown). These ratios are presented in the bar graph of Figure 2, panel A. The TS/CHCl_3 _ratio was significantly higher in DMSO samples than in TCDD samples, indicating that the latter extracts contained less total lipid.

TS predominantly represented membrane constituents and triglycerides. Membranes are thought to be relatively constant in terms of composition. Since membrane constituents such as phospholipids (PL) are easily detected in NMR-spectra, it was assumed that generic phospholipid signals (such as signals 18 and 20 in Figure 1; backbone signal for non-lyso-PL and lyso-PL) could be used as an internal measure for the amount of membrane and thus for the number of cells. No specific signals representing lyso-PL could be detected in the NMR spectrum, indicating that this species makes a negligible contribution to the total phospholipids. This was corroborated by other known membrane constituents including free cholesterol and polyunsaturated fatty acids, which had constant ratios to the phospholipids (vide infra). Normalization using a PL signal as an internal standard gave excellent results when the spectra were compared manually and when the PCA results were considered. Since a PL signal is an internal standard, it will reflect directly potential differences in biological replicate flasks and may reflect possible small differences in sample handling; therefore this normalization method is better than using an external standard such as a cell counting average obtained from additional cell culture flasks. A better normalization allows for the detection of smaller differences in concentrations of metabolites.

The ratio between the intensity of the PL signal (signal 20 in Figure 1) and CHCl_3 _(PL/CHCl_3_), presented in Figure 2, panel B, was used to normalize the data before multivariate analysis (MVA) was carried out. DMSO control extracts contained more phospholipids, and therefore more cells, than TCDD extracts (Figure 2). The difference in the number of cells between TCDD-exposed and control samples was significant for passage numbers 30, 7 and 11. For passage numbers 11b and 17, the differences in cell numbers did not reach significance.

When scaling on PL signals (Figure 1), the signal corresponding to the bis-allylic protons of long chain polyunsaturated acyl groups (PUFA; signal 14 in Figure 1) at 2.84 ppm was not changed, indicating unaltered levels of long chain PUFAs upon TCDD treatment. Furthermore, the signal relating to free cholesterol (signal 6 in Figure 1) was not altered. Long chain PUFAs and free cholesterol are important components of membranes and it was expected that these components would correlate with membrane-related phospholipid signals.

Representative ^1^H NMR spectra replicates of apolar extracts of HepG2 cells scaled on the PL signal are overlaid in Figure 1, and the assignment of the signals is given in Table 1. Figure 1 demonstrates an excellent overlapping of biological replicates of controls and of treated cell extracts. The main differences observed between the treated and non-treated cell extracts were related to triglyceride signals.

**Table 1:** Assignment of the ^1^H NMR spectral signals of the apolar fraction.

Peak number	Chemical shift (ppm)	**Assignment**^**a**^
1	0.68	Total cholesterol C^18^**H**_**3**_
2	0.85/0.86	Total cholesterol C^26^**H**_**3**_/C^27^**H**_**3**_
3	0.86-0.91	Acyl groups C**H**_**3 **_(all except n-3)
4	0.92	Total cholesterol C^21^**H**_**3**_
5	0.98	n-3 acyl groups C**H**_**3**_
6	1.01	Free cholesterol C^19^**H**_**3**_
7	1.02	Esterified cholesterol C^19^**H**_**3**_
8	1.06-1.19	Multiple cholesterol protons
9	1.20-1.38	Acyl groups -(C**H**_2_)n-
10	1.50-1.65	Acyl groups -OCO-CH_2_-C**H**_2_- and **H**_2_O
11	1.79-1.88	Multiple cholesterol protons
12	1.97-2.10	Acyl groups -C**H**_2_-CH=CH-
13	2.24-2.38	Acyl groups -OCO-C**H**_2_-
14	2.72-2.88	Acyl groups =HC-C**H**_2_-CH=
15	3.28-3.38	Choline N(C**H**_3_)_3_
16	3.90-4.02	Glycerophospholipids N-C**H**_2_
17	4.10-4.32	Glycerol backbone -C**H**_2_OCOR
18	4.33-4.43	Phosphatidylcholine PO-C**H**_2_
19	4.50-4.66	Esterified cholesterol C^3^**H**
20	5.17-5.24	Glycerophospholipid backbone >C**H**OCOR
21	5.24-5.28	Glycerol backbone >C**H**OCOR
22	5.29-5.43	Acyl groups -C**H**=C**H**-

Numbers correspond to those in Figure 1.

^a^Protons assignment based on [81]

**Figure 3:**
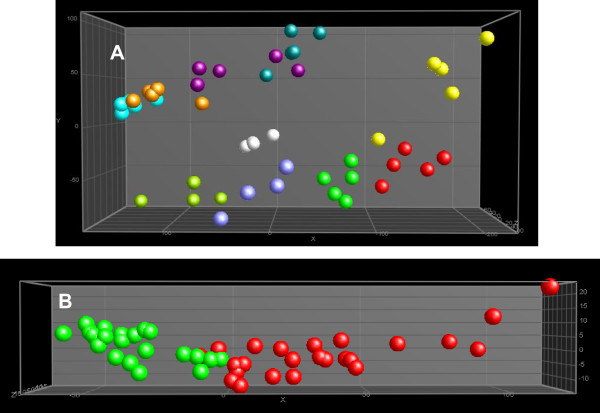
**PCA plots (after ANOVA p < 0.01) of the apolar fraction of HepG2 analyzed by ^1^H NMR and pre-processed and aligned using a program developed in-house.** A: Spheres with the same colour are technical replicates of the same sample per passage number. DMSO30: green; TCDD30: red; DMSO7: lila; TCDD7: yellow; DMSO11b: light blue; TCDD11b: dark blue; DMSO17: orange; TCDD17: purple; DMSO11b: light green; TCDD11b: white. B: Samples re-grouped with regard to treatment (irrespective of passage number). DMSO: green; TCDD: red.

NMR data were pre-processed and aligned using a program developed in-house [32,33]. The aligned fingerprint data were normalized using the PL/CHCl_3 _ratio and exported in the form of spreadsheets for further multivariate analysis. Figure 3A presents a PCA plot (after pre-selection using ^2^Log transformation and an ANOVA where p < 0.01) and demonstrates that the biological replicates of each sample were clustered (with or without ANOVA pre-selection; data not shown). It is remarkable that the passage number had an important effect, reflecting the difference in the magnitude of the effect of TCDD.

An alternative method of mining these normalized data sets is to perform a PCA after consecutively doing a ^2^Log transformation, a regrouping towards treatment (TCDD) and control (DMSO), and an ANOVA (p < 0.01) (Figure 3B). From the underlying peak loadings (additionally surviving a Bonferroni correction) responsible for the separation of samples in this PCA, it is possible to create a list of resonance positions that contribute significantly to the observed separation between the apolar fractions of cells exposed to TCDD and controls. The expected decrease in triglyceride content, as observed in Figure 1 (signal 17), was the major difference between the treated and non-treated samples (average ratio TCDD: DMSO = 0.5). This effect, clearly observed during visual inspection of the NMR spectra, differed significantly in the five experiments conducted. Furthermore, the concentration of cholesterol ester was decreased (average ratio TCDD: DMSO = 0.7) after TCDD treatment (see Figure 1 signal 7). The decreases in triglyceride and cholesterol ester content were accompanied by a decrease in the intensity of the signals related to unsaturated acyl groups (signals 12, 14 and 22 in Figure 1) due to allylic, bis-allylic and olefinic protons, respectively (average ratio TCDD: DMSO = 0.75).

**Figure 4:**
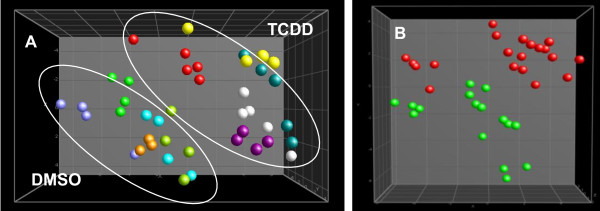
**PCA plots (after ANOVA p < 0.01) after normalization of the apolar fraction of HepG2 cells analyzed by GC-MS**. A: Spheres with the same colour are technical replicates of the same sample per passage number. For denotation of the colours, see legend Figure 3. B: Samples re-grouped with regard to treatment (irrespective of passage number). DMSO: green; TCDD: red.

The NMR data suggested that fatty acid composition changed as a result of TCDD exposure. Therefore, a GC-MS analysis of the fatty acid composition was performed on the apolar fraction of the cells. Consistent with the ^1^H NMR results, there was a difference in the apolar fractions of cells exposed to TCDD and DMSO with regard to the lipid content. Among the methyl esters of fatty acids that were identified, the arachidonic (AA, C20:4, n-6) and docosahexaenoic (DHA, C22:6, n-3) PUFAs had the same profile within the samples. The NMR data demonstrated that long chain PUFA content correlates highly with PL. Therefore, samples were normalized on the basis of the sum of the AA and DHA signals. This normalized dataset was subjected to MVA (^2^Log transformation, ANOVA with p < 0.01, followed by a PCA; see Figure 4A). The PCA plot obtained was similar to that obtained for ^1^H NMR.

In order to extract the most significant differences between the exposures, the samples were re-grouped with regard to the treatments and re-analyzed (PCA, after an ANOVA with p < 0.01; see Figure 4B and in Additional File 1, Table S1).

The selection of peaks underlying the largest differences (and additionally surviving a Bonferroni correction) between the treatments were identified. Table 2 presents these fatty acids and their fold change and indicates that the content of several fatty acids decreased due to TCDD treatment. However, there was a slight increase in the proportion of saturated fatty acids with 17 (average ratio TCDD: DMSO = 1.2) and 18 (average ratio TCDD: DMSO = 1.3) carbon atoms, and an increase in the proportion of an unidentified C18:2 isomer (average ratio TCDD: DMSO = 1.3) (Table 2). The decrease in several unsaturated groups observed in the GC-MS data (Table 2) is in agreement with the NMR data.

**Table 2:** Apolar metabolites significantly affected by TCDD (p < 0.01) and average fold change ratio between treatments (TCDD vs. DMSO).

*GC-MS*	*Ratio TCDD/DMSO (RTD)*
C12:0	0.78
C14:0	0.72
C14:1_a_	0.70
C14:1_b_	0.29
C16:1 (n-6)	0.63
C16:1 (n-9)	0.62
C17:0	1.20
C18:0	1.27
C18:1 (n-9)	0.78
C18:1_c_	0.72
C18:2_a_	0.71
C18:2_b_	0.63
C18:2_c_	1.29
C18:2_d_	0.64
C18:2_e_	0.63
C20:1_b_	0.79
C20:2_a_	0.74
C20:2_b_	0.70
C20:3_a_	0.80
C20:3_b_	0.64
C22:2	0.61
C22:3_a_	0.73

^***1***^***H NMR***	***Ratio TCDD/DMSO (RTD)***

Total triglycerides	0.51
Total cholesterol ester	0.69

*Underscore letters indicate isomers (unidentified).

Individual passage number results are in Additional File 1 (Tables S3 and S4).

### The polar fraction

**Figure 5:**
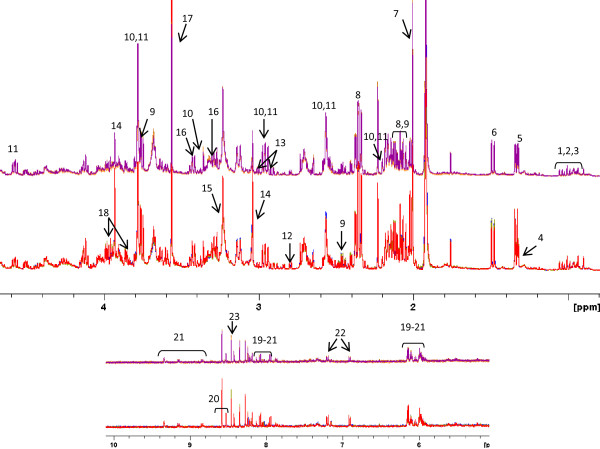
**^1^H NMR spectra overlaid (4-fold) of the polar fraction of HepG2 cells exposed to DMSO (below) and TCDD (above)**. The following metabolites are identified by using standards, from the literature and/or databases and the numbers of the signals correspond to those indicated in Table 3. 1&2: leucine and isoleucine; 3: valine: 4: threonine: 5: lactale; 6: alanine; 7: putrescine; 8: acetate; 9: N-acetyl-aspartate; 10: glutamate; 11: glutamine; 12: reduced glutathione; 13: oxidized glutathione; 14: acetone; 15: citric acid; 16: creatine and/or phosphocreatine; 17: choline derivatives; 18: taurine; 19: glycine; 20: serine; 21: nucleotides derived from uridine (UMP, UDP, UTP); 22: nucleotides derived from adenosine (AMP, ADP, ATP); 23: NAD+ and/or NADH; 24: tyrosine; 25: formate.

**Figure 6:**
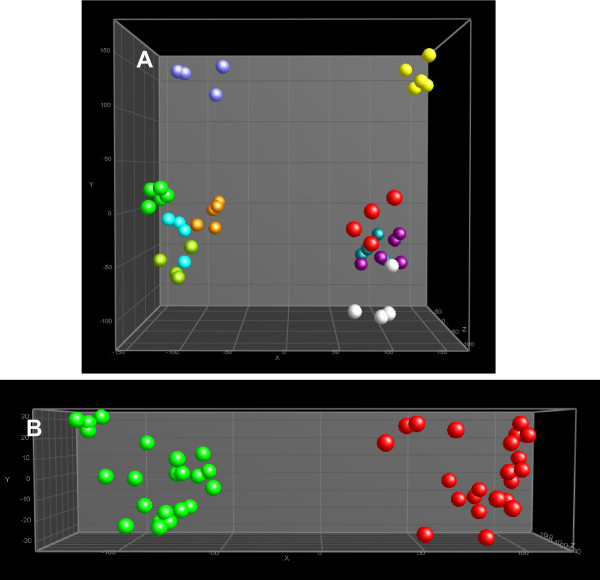
**PCA plots (after ANOVA p < 0.01) after normalization of the polar fraction of HepG2 cells analyzed by ^1^H NMR and pre-processed and aligned using a program developed in-house**. A: Spheres with the same colour are technical replicates of the same sample per passage number. For denotation of the colours, see legend to Figure 3. B: Samples re-grouped with regard to treatment (irrespective of passage number). DMSO: green; TCDD: red..

The ^1^H NMR spectra of polar fractions were normalized using the PL/CHCl_3 _ratio described above (obtained from the corresponding apolar fractions) and presented in Figure 2B. Representative normalized ^1^H NMR spectra replicates of polar extracts from HepG2 cells were overlaid (Figure 5) and the assignment of the signals is presented in Table 3. The normalized pre-processed and aligned NMR data were subjected to multivariate analysis and the output (PCA plot after ^2^Log transformation and ANOVA p < 0.01) is presented in Figure 6A. Differences between the polar fraction of cells exposed to TCDD and those exposed to the control can clearly be observed following the x-axis of the PCA. The y-axis represents the differences between the different passage numbers. To identify effects due to TCDD treatment, an ANOVA (p < 0.01) (after ^2^Log transformation) with regard to the treatment was performed, followed by PCA (Figure 6B). From the underlying peak loadings (additionally surviving a Bonferroni correction) responsible for the separation of samples in the PCA, it is possible to create a list of resonance positions that contribute significantly to the observed PCA separation.

**Table 3:** Assignment of ^1^H NMR spectral signals of the polar fraction.

Peak number	**Chemical shift (ppm) and multiplicity**^**b,c**^	**Assignment**^**a**^
1	0.96 (t)	Leucine
2	0.96, 1.05 (t)	Isoleucine
3	1.01, 1.04 (d)	Valine
4	1.32 (d)	Threonine
5	1.33 (d)	Lactate
6	1.48 (d)	Alanine
7	2.00 (s), 2.48 (dd)	N-acetyl-aspartate
8	2.08 (m), 2.34 (m)	Glutamate
9	2.12 (m), 2,44 (m), 3.76 (m)	Glutamine
10	2.18 (m), 2,57 (m), 2.98 (dd), 3.31 (dd), 3.78 (m)	Oxidized glutathione
11	2.18 (m), 2,57 (m), 2.98 (dd), 3.78 (m), 4.58 (dd)	Reduced glutathione
12	2.80 (dd)	Aspartate
13	2.55 (d), 2.67 (d)	Citrate
14	3.04 (s), 3.93 (d)	Creatine/Phosphocreatine
15	3.23 (s)	Choline derivatives
16	3.26 (t), 3.41 (t)	Taurine
17	3.56 (s)	Glycine
18	3.83 (dd), 3.98 (dd)	Serine
19	6.10 (d), 6.14 (d), 8.18-8.22 (ov), 8.52 (s), 8.58 (s)	Nucleotides derived from adenosine AXP (AMP, ADP and/or ATP)
20	5.95-5.99 (ov), 7.94-7.97 (ov), 8.09 (d)	Nucleotides derived from uridine UXP (UMP, UDP and/or UTP)
21	6.01-6.20 (ov), 8.12-8.20 (ov), 8.79-8.88 (ov), 9.08-9.36 (ov)	Nicotinamide adenine dinucleotides (NAD, NADH, NADP and/or NADPH)
22	6.99 (m), 7.20 (m)	Tyrosine
23	8.46 (s)	Formate

Numbers correspond with those in Figure 5.

^a^Protons assignment based on commercial standards, Human Metabolome Database [80] and on ref [82,83]

^b^s: singlet; d: doublet; dd: doublet/doublet; m: multiplet; ov: different overlapping signals

^c^Only observed signals are shown

The normalized NMR data indicated that the levels of several polar intracellular metabolites decreased due to exposure to TCDD (Table 4). However, the intensities of signals relating to taurine (average ratio TCDD: DMSO = 1.4), citrate (average ratio TCDD: DMSO = 1.5), reduced glutathione, GSH (average ratio TCDD: DMSO = 1.6) and oxidized glutathione, GSSG (average ratio TCDD: DMSO = 1.3) increased. The GSH/GSSG ratio increased by 18% due to TCDD exposure.

**Table 4:** Polar metabolites significantly affected by TCDD exposure.

Metabolite	**Change fold (**^**1**^**H NMR p < 0.01)**	Change fold (LC-MS p < 0.01)
Leucine, Isoleucine	0.69	0.64
Valine	0.72	0.69
Alanine	0.68	--
N-acetyl-aspartate	0.68	0.72
Glutamate	0.79	0.80
Glutamine	0.69	--
Glycine	0.72	--
Aspartate	0.66	--
Serine	0.65	--
Tyrosine	0.58	0.44
Proline	--	0.54
Tryptophan	--	0.79
Lactate	0.49	--
Spermidine	--	0.24
N1-acetyl-spermidine	--	0.59
Pantothenic acid	--	0.62
Creatine/Phosphocreatine	0.66	--
Propyonylcarnitine^b^	--	0.66
Butyrylcarnitine	--	0.58
Nucleotides (AXP and UMP)	0.3-0.6	--
UMP	--	0.71
AMP	--	0.67

Oxidized glutathione	1.26	1.31
Reduced glutathione	1.58	1.23
Taurine	1.37	--
Citrate	1.46	1.68
UDP-N-acetyl-galactosamine/glucosamine*	--	1.48

The fold changes are given as the ratio of the average content in TCDD and in DMSO. Individual passage number results are in Additional File 1 (Tables S5 and S6).

*Tentatively identified

**Figure 7:**
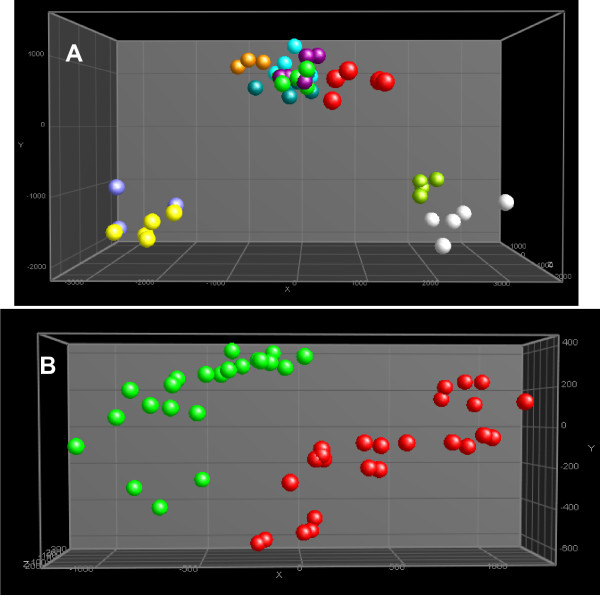
**PCA plots (after ANOVA p < 0.01) after normalization of the polar fraction of HepG2 cells analyzed by LC-ToF-MS and pre-processed and aligned using MetAlign (www.metalign.nl)**. A: Spheres with the same colour are technical replicates of the same sample per passage number. For denotation of the colours, see legend Figure 3. B: Samples re-grouped with regard to treatment (irrespective of passage number). DMSO: green; TCDD: red.

The same extracts were analyzed by LC-MS in order to obtain more detailed information concerning the differences in polar metabolite levels due to treatment. The LC-MS data were pre-processed and aligned using MetAlign, the program developed in-house [34]. The aligned fingerprint data, in the form of generated spreadsheets, were normalized using the PL/CHCl3 ratio and subjected to ^2^Log transformation and ANOVA with p < 0.01 followed by PCA (Figure 7A). The separation between samples was dominated by passage number rather than by treatment. In order to determine the effect of TCDD on the polar fraction of the cells, the samples of this dataset were re-grouped with regard to treatment, and an ANOVA (p < 0.01) followed by a PCA was performed after ^2^Log transformation (Figure 7B). From the peak loadings (additionally surviving a Bonferroni correction) responsible for the separation of samples in this PCA, it is possible to create a list of masses that contributed significantly to the observed PCA separation. This list of masses was screened for molecular ions that showed at least a 1.2 fold change due to the treatment. Table 4 lists the metabolites and their fold change, as determined on the basis of fragmentation data from LC-nanomate-Orbitrap experiments. Table 5 presents the exact experimental masses of these metabolites, together with their obtained fragments. A few metabolites remained unidentified.

**Table 5:** Masses and fragments of the masses of polar metabolites identified by means of LC-nanomate-Orbitrap.

Compound	**Parent mass(M+1)**^**1**^	Neutral formula	**Deviation (mDa)**^**1**^	**Fragments**^**1**^
Proline	116.07046	C_5_H_9_NO_2_	-0.145	70.0649

				86.0961
Leucine/Isoleucine	132.10191	C_6_H_13_NO_2_	0.005	69.0696

				72.0805
Spermidine	146.16503	C_7_H_19_N_3_	-0.144	112.1117

				158.0443
N-acetyl-aspartate	176.05542	C_6_H_9_NO_5_	0.091	134.0444
				88.0390

				165.0542
Tyrosine	182.08118	C_9_H_11_NO_3_	0.010	136.0754
				119.0489

				72.0806
Acetyl spermidine	188.17580	C_9_H_21_N_3_O	0.061	17.1019
				171.1487

				188.0702
L-tryptophan	205.09717	C_11_H_12_N_2_O_2_	0.016	146.0597
				118.0648

				193.0339
Citric acid (NH4^+^)	210.0606	C_6_H_8_O_7 _(+NH_3_)	0.002	175.0233
				129.0179

				173.0804
Propionylcarnitine	218.13869	C_10_H_19_NO_4_	0.005	155.0699

				202.1070
Pantothenic acid	220.11792	C_9_H_17_NO_5_	-0.029	184.0963
				90.0547

				100,0754
Unknown^2^	230.18622	C_11_H_24_O_2_N_3_	-0.084	183.1121
				213.1520

				173.0802
Butyrylcarnitine	232.15416	C_11_H_21_NO_4_	-0.175	85.0281

Unknown^2^	250.03784	C_6_H_12_O_3_N_4_P_2_	-0.075	---

Unknown^2^	258.11020	C_8_H_20_O_6_NP	0.100	104.1067
		C_7_H_14_ON_8_P	0.104	

				179.0414
				162.0216
Glutathione reduced	308.09103	C_10_H_17_N_3_O_6_S	-0.052	144.0110
				116.0161

UMP	325.04318	C_9_H_13_N_2_O_9_P	0.037	97.0281

		C_10_H_14_N_5_O_7_P	0.001	136.0615
AMP	348.07036			119.0349

				296.0650
Unknown	425.10794	---	---	350,0753

UDP-N-acetyl-galactosamine/glucosamine^2^	608.08866	C_17_H_27_N_3_O_17_P_2_	-0.186	405.0079
				204.0861

Glutathione oxidized	613.15906	C_20_H_32_N_6_O_12_S_2_	-0.178	---

^1^Experimental value obtained in our measurements

^2^Tentatively assigned formula(e) and/or identification

There were significant increases in citric acid (average ratio TCDD: DMSO = 1.7) and in GSSG (average ratio TCDD: DMSO = 1.3) and GSH (average ratio TCDD: DMSO = 1.2) contents in TCDD treated samples, in general agreement with the ^1^H NMR data. However, the GSH/GSSG ratio as determined from LC-MS data was reduced, in contrast to the NMR data. Generally, NMR data are more quantitative and reliable; furthermore, the LC-MS data were acquired at a later date than the NMR data. In a follow-up NMR study on the stability of the sample, it was observed that the intensity of the GSH signal decreased over time during storage whereas the intensity of the GSSG signal increased (see Figure S1 in Additional File 1). No other metabolic changes were observed.

This reaction is known to be dependent on pH (i.e. more prominent at higher pH). Therefore storage conditions such as pH are important. NMR samples were measured directly after adding phosphate buffer to the freshly made extracts. In this study the UPLC-TOF/MS samples were stored at -20°C without the phosphate buffer and therefore under more acidic conditions (pH ca. 5 as a result of the extraction conditions). The maximum storage time before UPLC-TOF/MS measurement was 4 months. Under these storage conditions still some conversion to of GSH to GSSG was present as deduced from our experiments.

## Discussion

The main aim of this study was to demonstrate the feasibility of using metabolomics on intracellular compounds of *in vitro *cell systems such as HepG2 cells, as an alternative approach to studying the effects of toxicants. We have demonstrated that reproducible results can be obtained and that differences between controls from different experiments can be subjected to statistical analysis, but that caution is required with regard to repeatability of experiments; generally, the same effects are observed in different experiments but their magnitudes can differ. However, there is a consistent effect (due to TCDD in this study) on metabolic profiles using an untargeted approach. The changes in metabolite concentrations are minimal and can vary because of the effect of the treatment on cell numbers. Therefore, normalization (using PL and PUFA), as described above, is crucial; so is having enough biological replicates to ensure the accuracy of the data. Without normalization or sufficient biological replicates, several of the metabolic changes may not have been detected. In apparent contradiction to the normalization using PL, it has been reported that TCDD induces the expression of the phospholipase A2α (PLA2α) gene in mouse hepatoma Hepa-1c1c7 [35]. However, in this HepG2 study, degradation of PL was not evident with regard to total PL. Furthermore, a parallel study using transcriptomics did not provide evidence of an effect of TCDD on PLA2α gene expression in HepG2 cells; this supports the present findings for HepG2 cells and supports the validity of the chosen normalization method.

To substantiate the usefulness of the untargeted metabolomics methodology, the observed changes in metabolite levels were interpreted in the light of published data concerning the effect of TCDD. Ultimately, this was the validation of the procedures and protocols developed in this study. In general, a decrease or increase of an intracellular metabolite concentration is not necessarily an indicator of flux. Actual metabolite levels depend on how biochemical pathways/processes are regulated, how metabolites in pathways interact and how fast and in which directions reactions occur (kinetics). Cells tend to balance their metabolism after perturbation in order to maintain homeostasis. Therefore, it is likely that only changes in the main pools of substrates in the cell are measured when it is exposed to a toxicant. Inhibition/activation of a pathway may lead to increased or decreased levels of relevant pools of compounds. However, it is often difficult to determine whether inhibition or activation is relevant by observing metabolite concentrations at one time point. As discussed in more detail below, TCDD is known to have an effect on the proliferative capacity, metabolism and antioxidant status of the cell.

### Effect on cell proliferation

Membrane constituents such as PL (Figure 2B) and free cholesterol are present at lower levels in extracts obtained after treatment with TCDD because there are fewer cells in the culture, and are indicative of decreased proliferation during exposure; the anti-proliferative effect of TCDD has been described previously [26]. The lower levels of nucleotides in treated extracts indicate a change in metabolites known to be involved in DNA synthesis (Table 4).

Spermidine and N1-acetylspermidine were also found in lower levels after TCDD treatment. Polyamines have important functions in cell proliferation, differentiation and apoptosis, and they are essential for hepatic growth and regeneration [36]. Polyamines can be synthesized in liver from agmatine (formed by decarboxylation of arginine, via arginine decarboxylase) [37], but ornithine decarboxylase (ODC) is the main enzyme responsible for polyamine biosynthesis. ODC is involved in the conversion of ornithine into putrescine, the precursor of other polyamines [38,39]. Their catabolism, on the other hand, is regulated by the enzymatic action of the polyamines spermidine/spermine N1-acetyltransferase (cSAT) and diamine oxidase [40].

ODC is induced during conditions where there is enhanced gene activation and tissue growth. It has been reported that ODC induction is inhibited by TCDD [38,39].

Therefore, it is possible that induction of polyamine synthesis in cultured HepG2 cells was inhibited by TCDD, resulting in the low content of spermidine.

### Effect on metabolism

Changes in metabolic profiles due to TCDD treatment of HepG2 cells indicates major changes in the general metabolism of these cells, which involves fatty acids, amino acids and nucleotides. This is reminiscent of wasting syndrome [21,41-43].

The decreases in triglycerides, cholesterol ester and unsaturated fatty acids observed in the ^1^H NMR analysis of the apolar fraction, together with the decreases in several fatty acids observed in the GC-MS data, indicate that under the conditions used in this study, TCDD affected lipid metabolism.

Several *in vivo *studies [44-46] have demonstrated that TCDD reduces the activity of enzymes involved in the synthesis of *de novo *fatty acids (such as fatty acid synthase, FAS and acetyl CoA carboxylase, AAC) and in cholesterol biosynthesis (such as 3-hydroxy-methylglutaryl-CoA synthase) or the expression of genes encoding these enzymes [41,42,47-49]. This effect, which was also observed in experiments using mouse embryo fibroblasts [50], supports the results presented here. In studies carried out *in vivo *[49] and *in vitro *[50], it has been reported that the effect of TCDD on the gene expression of enzymes involved in the *de novo *synthesis of fatty acids is mediated by AhR.

Using GC-MS analysis it is possible to determine the nature of the fatty acids that are most affected by exposure to TCDD (Table 2). The levels of the majority of fatty acids decreased upon treatment. However, the contents of heptadecanoic (C17:0) and octadecanoic, i.e. stearic (C18:0), acids were increased after exposure. The increased percentage of saturated and the decreased percentage of mono-unsaturated fatty acids could be due to the decreased expression of stearoyl-CoA-desaturase (SCD1), as demonstrated in mouse embryo fibroblasts after TCDD exposure [51]. SCD1 is the rate limiting enzyme in the biosynthesis of mono-unsaturated fatty acids and catalyzes the introduction of the *cis *double bond in the Δ9 position of acyl CoA substrates [52]. Therefore, inhibiting the expression of SCD1 using TCDD would partially explain the increased ratio between saturated/monounsaturated fatty acids observed in this study.

Pantothenic acid and AMP are metabolites found in lower concentrations after exposure to TCDD (Table 4). Pantothenic acid and AMP are two of the three precursors for coenzyme A (CoA) synthesis. In general, during fatty acid degradation and synthesis, CoA transports fatty acids as acyl groups through repetitive degradative or synthetic cycles.

Furthermore, decreases in the content of propionyl- and butyryl-carnitines were observed when cells were exposed to TCDD. These short chain acyl-carnitines are metabolic products of the reaction of acyl CoA and carnitine, which is mediated by transferases in the mitochondria [53]. Formation of butyryl- and propionyl-carnitines is favored when butyryl and propionyl-CoA accumulate in the mitochondria owing to an increased acyl-CoA/CoA ratio. In the case of propionyl-CoA, an increase in the catabolism of amino acids such as valine, isoleucine and - indirectly - threonine and methionine also favors its formation [54]. Changes in these carnitine derivatives indicate that mitochondrial activity and transport across mitochondrial membranes is affected by CoA modulation and/or beta-oxidation of fatty acids.

The level of citrate increased, whereas the level of lactate decreased, after exposure. Citrate is a feedback inhibitor for glycolysis at the level of phosphofructokinase. Lactate is a product of the conversion of pyruvate by lactate dehydrogenase. These two metabolite changes can indicate that glycolytic activity is lower because of an influx of acetyl-CoA, for instance due to fatty acid beta-oxidation. This is in agreement with the breakdown of triglycerides and degradation of fatty acids, and changed activity in mitochondria (see above).

Together, these metabolic effects are reminiscent of a wasting syndrome in which fatty acid metabolism and energy metabolism (glycolysis, TCA cycle) are affected, and the amino acid and nucleic acid pools are reduced. These latter effects could contribute to the observed decrease in cell proliferation. However, it is not clear from the data whether beta-oxidation increased or if fatty acids were transported out of the cell. In adipose tissue, TCDD mobilizes fatty acids towards the plasma [41,42,46,55,56]. An increase in amino acids circulating in plasma owing to TCDD exposure has previously been reported [57]. Therefore, the data could imply a shut-down of the cells followed by the export of several metabolites.

Several *in vivo *studies demonstrate an accumulation of lipids in the liver [21,46,55,58], although the effects of TCDD vary depending on the dose, exposure-time or test animal used [41,44,45,59]. An increase in liver lipid content is in contrast to the results found in this research. However, in the published studies, a loss in body fat is demonstrated, as is an increase in the serum lipid content [41,42,46,55,56]. The increased serum lipid content could increase uptake and accumulation of extrahepatic lipids in liver [46,60]. This would explain the occurrence of a fatty liver *in vivo*, and the differences in terms of lipid content between *in vivo *and *in vitro *studies.

### Effect on antioxidant status

NMR and LC-MS spectra demonstrated an increase in reduced (GSH) and oxidized (GSSG) glutathione content in samples treated with TCDD (Table 4). However, it has been reported that GSH content decreases after exposure to TCDD [61]. Furthermore, from the ^1^H NMR data, it was observed that the GSH/GSSG ratio increased ca. 20% with TCDD exposure. This ratio, considered a parameter for measuring the oxidative status of a cell, has been reported to decrease in several *in vivo *studies as a consequence of exposure to TCDD [23,61]. However, data are variable and some authors have reported an increase in the GSH/GSSG ratio after mice were treated with TCDD [22].

GSH acts as a nucleophilic "scavenger" of numerous compounds and their metabolites via enzymatic and chemical mechanisms (converting electrophilic centers to thioether bonds) and as a substrate in the glutathione peroxidase (GPx)-mediated reduction of lipid hydroperoxides and of H_2_O_2 _to water [62,63]. As a consequence of this reaction, GSH is oxidized to GSSG, which is in turn recycled back to GSH via glutathione reductase (GR). Therefore, any effect on the activity of GPx, GR or both could affect the ratio between GSH and GSSG. Several *in vivo *[61] and *in vitro *[64,65] studies have described the inhibition of GPx activity in liver due to TCDD treatment. Inhibition of the activity of this enzyme could theoretically lead to an increase in the content of GSH and in the GSH/GSSG ratio, as observed herein. However, it has been reported that the activity of GPx in liver mitochondria of mice exposed to TCDD is increased [22], but increased activity of GR in liver mitochondria was also evident, possibly explaining the increased GSH/GSSG ratio following dioxin treatment. Some studies have demonstrated that TCDD increases the activity of GR in liver cells [66], whereas other authors describe the opposite effect [64].

In relation to the synthesis of GSH, Boverhof and co-authors [41] demonstrated up-regulated expression of the genes for enzymes involved in the synthesis of GSH (such as glutamate-cysteine ligase, Gclc, and glutathione synthase, Gss) after rats and mice were exposed to TCDD. Increased synthesis of GSH could explain the results. Boverhof and co-authors [41] described a down-regulation of the genes associated with the metabolism of glutamate and glycine, which are building blocks of GSH. They suggested that this down-regulation could be an adaptation of the cell to conserve these amino acids for increased glutathione synthesis.

### Other effects associated with TCDD treatment

Creatine is decreased in cells as a result of TCDD treatment (Table 4). In relation to this, Boverhof and co-authors [41] found that genes expressing guanidinoacetate N-methyltransferase (GAMT), one of the key enzymes in the biosynthesis of creatine, are down-regulated in liver after TCDD administration to rats. It has been reported that GAMT deficiency is associated with a reduction in body weight owing to reduced body fat mass [67], which could be related to the aforementioned *in vivo *effects of TCDD exposure.

The content of taurine, a non-protein sulfur-containing β-amino acid, is increased after the exposure of cells to TCDD. Taurine plays an important role in several biological processes including anti-oxidation, detoxification, membrane stabilization and maintenance of osmolarity [68]. Moreover, it has been demonstrated that there is a relationship between taurine and lipid metabolism [69,70]. Taurine lowers hepatic triglyceride concentration, reduces the synthesis of cellular cholesterol ester and elevates hepatic free fatty acids. These effects are in agreement with the results of the present study for HepG2 cells exposed to TCDD. TCDD down-regulates cysteine dioxygenase (CDO) [41,42,71,72], the first enzyme in the conversion of cysteine to taurine. This seems to conflict with the observation of higher taurine levels after exposure to TCDD, but since the regulation of taurine concentration is unknown, perhaps the increased concentration of taurine after exposing cells to TCDD is related to other mechanisms.

An increased signal (average ratio TCDD: DMSO = 1.5) tentatively assigned to uridine diphosphate-N-acetylgalactosamine (UDP-NAcGal) and/or uridine diphosphate-N-acetylglucosamine (UDP-NacGlu) was observed after TCDD exposure (Table 4).

UDP-NAcGal and UDP-NacGlu are N-acetylhexosamines involved in several biosynthetic reactions including O-glycosylation of proteins on serine and threonine residues. O-linked glycosylation involves the transfer of the N-acetylhexosamine group from the UDP-acetylhexosamine, which is catalyzed by glycosyltransferases [73,74].

Lower activity of these enzymes could theoretically produce an increase in the levels of UDP-N-acetylhexosamines. In agreement with this, decreased expression of GALNT1, the gene encoding the UDP-N-acetylgalactosamine transferase, was observed by Kim and co-authors [75] after HepG2 cells were exposed to TCDD.

Changes in the intracellular levels of UDP-N-acetylhexosamines, as observed in this study after exposure to TCDD, may indicate that protein O-glycosylation is affected. Altered expression levels of glycosylated protein have been described after TCDD exposure *in vitro *[76].

We have no explanation for the decrease in concentration of N-acetyl aspartate. This metabolite is normally expressed at high concentrations in the brain and has been implicated as an osmolyte [77] with the potential to bind to calcium ions or metal ions [78].

## Conclusions

The present study demonstrates that untargeted profiling of the polar and apolar extracts of *in vitro *cultured HepG2 cells using various analytical techniques is a feasible approach to studying the effect of a toxicant, TCDD, on the cell metabolome. In the metabolomics methodology used, sample protocols and handling were minimized to enhance reproducibility. The results are compatible with previously well-documented effects of TCDD, *in vitro *and *in vivo*. This serves to validate the metabolomics results produced in this study. This combination of cell culture and metabolomics technology (together with other -omics techniques) can contribute to reducing the number of animals required for toxicity studies.

This study is an example of how complementary analytical techniques (NMR, GCMS, LCMS) can be used on the same samples and provide complementary data. The untargeted nature of the experiments aids in finding differences in profiles, which are then subjected to identification approaches. As discussed, normalization and sufficient biological replicates (and repeated experiments) are essential for reliability (in terms of statistical analysis) in detecting relatively small differences in analyte concentrations.

Maintaining homeostasis could be the driving force in cells responsible for keeping concentration differences small. The effects are predominantly centered on storage pools of metabolites.

## Methods

### Chemicals

Minimal essential medium (MEM glutamax), non-essential amino acids, sodium pyruvate, penicillin/streptomycin and fetal bovine serum were purchased from Gibco BRL (Breda, The Netherlands). Ammonium acetate (NH_4_Ac), sodium hydroxide (NaOH), sodium chloride (NaCl), dipotassium hydrogenphosphate (K_2_HPO_4_), monopotassium hydrogenphosphate (KH_2_PO_4_), boron trifluoride (BF_3_), EDTA and deuterated chloroform (CDCl_3_), deuterium oxide (D_2_O) and deuterated methanol (CD_3_OD) were obtained from Merck (Darmstadt, Germany); methanol (MeOH) and iso-octane from Biosolve (Valkenswaard, The Netherlands); DMSO and TMSP from Sigma-Aldrich (St. Louis, MO, USA) and TCDD (CAS no. 1746-01-6) from Cerilliant (Round Rock, TX, USA). All chemicals and solvents were purchased in the highest purity available. Ultra-pure water was obtained using the PureLab equipment from Rossmark (Ede, The Netherlands).

### Cell culture treatment and disruption

HepG2 cells were obtained from the ATCC (Rockville, MD) and cultured in MEM glutamax supplemented with 1% non-essential amino acids, 1% sodium-pyruvate, 1% penicillin/streptomycin and 10% fetal bovine serum (FBS). The cells were incubated at 37°C with 5% CO_2_. When the cell monolayer reached 80% confluency, the medium was replaced with fresh medium with or without TCDD. TCDD was administered to a final concentration of 10 nM by adding 60 μl of a solution of 2 μM of TCDD in DMSO to 12 ml of medium. In the control treatments, cells were exposed to 12 ml of medium to which 60 μl of DMSO had been added. The final concentration of 10 nM TCDD was sub-cytotoxic and has been used in previous omics studies [1,15] The treatment of HepG2 cells with 10 nM TCDD and the vehicle control (DMSO) was terminated after 48 h (as in previous studies) by washing the cells several times with ice-cold 0.9% NaCl in ultra-pure water and disrupting them by osmotic shock with ice-cold ultra-pure water. The cells were harvested using a cell scraper and subsequently treated ultrasonically to ensure total disruption. The TCDD effect on the cell metabolome was studied in five independent experiments, using five different passage numbers (p+7, p+11a, p+11b, p+17 and p+30). All cells, except those in p+11b, originally came from the same cryogenic vial; those in p+11b were obtained from a second vial of frozen cells in order to check whether there were differences between the same passage numbers from different vials (p+11a *vs*. p+11b). Four independent biological replicates (i.e. tissue culture flasks) were examined in each experiment.

A separate study - prior to this one - with additional replicates was done to establish the suitability of the normalization approach based on PL signals. This study involved cell counting. In short, the cells were detached from the flasks by trypsinization and counted using a hemocytometer (Neubauer chamber). The results are given in Table S2, Additional File 1.

### Extraction of the apolar and polar fractions

The metabolomics study was performed on two different fractions of the cells, one apolar fraction containing the apolar metabolites (membranes and intracellular lipids), and one polar fraction, containing the polar and semi-polar intracellular metabolites. To extract these, the disrupted cells were centrifuged (4°C) and the pellet (apolar fraction) and supernatant (polar fraction) were separated and processed.

#### Apolar fraction

The pellet was re-suspended in 1 M NH_4_Ac and freeze-dried. Once dried, the residue was extracted three times with 1 ml of CDCl_3_, after which the organic solvent was evaporated under N_2 _flow. The dried extract was dissolved in 1 ml of CDCl_3_; an aliquot (0.6 ml) was used for NMR analysis and the remaining sample was stored at -20°C for GC-MS analysis.

#### Polar fraction

The supernatant containing the polar intracellular metabolites was freeze-dried. The pellet was re-suspended in 1 ml of 100% CD_3_OD, dried with N_2 _and re-suspended in 50% CD_3_OD/50% D_2_O. The solution was centrifuged to remove the proteins (in the pellet) and the supernatant was dried under N_2 _flow. The dry residue was dissolved in 1 ml of D_2_O. Some of the sample (350 μl) was stored at -20°C for UPLC-TOF/MS analysis. To the remainder, 1 M phosphate buffer (90:10; v: v; pH = 7) in 99.95% D_2_O was added and used for NMR analysis.

### Analysis of the polar and apolar fractions

#### NMR analysis

The samples were analyzed by NMR just after extraction. Some of these samples were also used for a follow-up study with the aim of determining the stability of the different metabolites over time. These samples were stored at -20°C in phosphate buffer (pH = 7) for 9 months.

The ^1^H NMR spectra were recorded at 400.13 MHz at 300.0 (± 0.05) K on a Bruker Avance 400 narrow bore using a 5.0-mm probe. The spectrometer settings were 2s relaxation delay; 1024 scans for the polar fraction and 128 scans for the apolar fraction, with four dummy scans in all cases; a spectral width of 5000 Hz; a 60 degree pulse; acquisition in 16 K data points. Prior to data analysis, the raw NMR data were subjected to a squared sine bell filter (shifted 1/2 pi), zero-filling to 128 K data points, Fourier transformation and phase correction.

NMR raw data and the corresponding metadata are provided as an additional file (see Additional file 2).

#### GC-MS analysis

##### Derivatisation

A miniature scale BF3-mediated methanolysis method for derivatizing fatty acids (for GC-MS) was used. In brief: 132 μl of the apolar fraction was dried under nitrogen. To this dry extract, 40 μl of 0.5 N NaOH in MeOH was added. The capped vial was placed in a pre-heated oven at 65°C for 30 minutes after which 50 μl of BF3 (20% in methanol) was added. After closing it again, the vial was put in the oven for three minutes at 65°C; 0.2 ml of iso-octane was added. The vial was again closed and put in the oven for two minutes at 65°C. After cooling, the volume was adjusted to 1 ml with a saturated NaCl solution. This was shaken firmly and the content was left to separate into two phases. The vial was centrifuged at 2800 rpm and the upper phase was carefully collected. For GC-MS analysis, 75 μl of the iso-octane phase was transferred into a new vial and kept at -20°C pending analysis.

##### GC-MS

A Trace GC 2000 series gas chromatograph interfaced with a Trace MS Plus mass spectrometer (Thermo Finnigan, San Jose, CA) was used for GC/MS analysis. Two μl of sample was injected using a PTV-Splitless injection. For the separations, a RTX-5 column (length 10 m, internal diameter 0.18 mm, stationary phase film 0.20_m; Alltech, Breda, The Netherlands) was used with helium as the carrier at a constant flow of 1.3 ml/min. The GC temperature program was: 2 min at 80°C; increase temperature at 5.5°C min^-1 ^to 185°C followed by 3.5°C min^-1 ^to 290°C. This latter temperature was kept for 12 min and afterwards it decreased by 120°C min^-1 ^to 150°C. The GC-MS interface temperature was 280°C; the source temperature was 200°C. Mass scanning in EI (electron impact) mode was carried out for the range of 50-500 m/z at a scan time of 0.4 seconds. The detector voltage was set to 500 V and the setting of the EI ionization source was 70 eV. All data were collected consecutively in one analysis series to minimize chromatography differences and the injection sequence was randomized according to Vos and co-authors [2].

Metadata for GS-MS files are provided as an additional file (see Additional file 2). Raw GS-MS data will be provided by a download link on request

#### UPLC-TOF/MS analysis

The fraction containing the polar metabolites was stored for a maximum of 4 months at -20°C (pH = 5) before analysis by UPLC-TOF/MS. UPLC-TOF/MS samples were diluted twice with D_2_O prior to analysis; formic acid was added to a final concentration of 0.1%. The injection sequence was randomized according to Vos and co-authors [2]. The analyses were performed on a LCT Premier LC-TOF-MS system (Waters, Milford, MA, USA) equipped with a dual spray electrospray source. The lock mass calibrant (leucine/enkephaline) was measured every 10 scans. The gradient was provided by an UPLC system (model Acquity, Waters) with a 150 mm × 2.1 mm UPLC BEH-C8 column with 1.7 μm particles (Waters).

The mobile phase consisted of water, acetonitrile and formic acid (A:100/0/0.2 and B: 0/100/0.2). Gradient elution was performed at 0.4 ml min^-1^. The initial eluent composition, 100% A, was held for one min after which the composition was changed to 85% A and 15% B in 14 min. Afterwards, the composition of B was increased to 30% in 10 min and subsequently increased again to 90% in three min; this composition was maintained for five min. The injection volume was 10 μl. The effluent of the LC system was interfaced directly with the TOF-MS, which was operated in positive mode polarity. A stable spray was obtained with a capillary voltage of 2.5 kV, a source temperature of 120°C and desolvatation temperature of 350°C. The desolvatation and cone gas flows were 600 and 50 L h^-1^, respectively. The cone voltage was 50 V. Spectra were collected in centroid mode from m/z 80 to 1500, with a scan duration of 0.2 s. Accurate masses were obtained after lock mass correction. The mass spectrometer was operated in W mode with the Dynamic Range Enhancement turned on and the resolution was 8500 (FWHM).

Metadata for LC-MS files are provided as an additional file (see Additional file 2). Raw LC-MS data will be provided by a download link on request."

#### LC-nanomate-Orbitrap analysis

##### Liquid Chromatography-Mass Spectrometry (LC-PDA-MS)

The system consisted of an Accela U HPLC system (pump and auto sampler) equipped with an Accela PDA with a one cm light Pipe flow cell (ThermoFisher Scientific) and coupled to a LTQ/Orbitrap hybrid mass spectrometer (Thermo Fisher Scientific) that was equipped with a chip-based nano-electrospray ionization source (Triversa NanoMate (Advion BioSciences). The sample (10 μl) was separated on a 150 mm × 2.1 mm UPLC BEH-C8 column with 1.7 μm particles (Waters). The flow rate was set at 0.4 ml min^-1^. Isopropanol (60 μl min^-1 ^of 100%) was added between PDA and NanoMate via a T-junction to the LC flow to ensure a stable nanospray. The sample was loaded with 100% eluent A (H_2_O/0.1% formic acid). The gradient was the same as that used for the UPLC-TOF/MS analysis. The PDA detector, placed between the analytical column and the NanoMate, was programmed to acquire data every second from 210 to 600 nm with a resolution of 1 nm and 2 Hz sampling rate. The NanoMate source was operated in the positive ionization mode with a HD_A_384 chip with a spray voltage of 1.4 kV and 0.4 psi N_2 _gas. The NanoMate was used in the LC coupling mode with fraction collection. The total flow (460 μl/min) was split using capillary tubing for MS spray (480 nl/min) and for fractionation (459.5 μl/min). Fractions were collected by the NanoMate from retention time 0.5 to 19.5 min in 3 s fractions (23 μl) in a 384 well plate (twin tec, Eppendorf). The plate temperature was set at 10°C. After collection, extra isopropanol (5 μl) was added. The plate was sealed to prevent evaporation of the fractions. These fractions were used in the MS^n ^experiments. Parallel to the fractionation, the MS spray from the NanoMate was used to record a full FTMS scan (m/z 80-1000) with resolution of 15.000 (at m/z 400). Automatic tuning was carried out on m/z 566.89. The full AGC target was set to 30000. The Orbitrap was externally calibrated in positive mode using sodium formate clusters in the range m/z 158.69-1110.79.

##### MS^n ^by direct infusion

The system consisted of a LTQ/Orbitrap hybrid mass spectrometer (Thermo Fisher Scientific) equipped with a chip-based nano-electrospray ionization source (Triversa NanoMate, Advion BioSciences). After manually selecting the masses of interest, recorded in the full scan MS, the fractions containing these masses were subjected to MS^n ^fragmentation in positive mode. Delivery of the sample to MS: The NanoMate used a pipette tip to take the sample from a selected well and infused this sample to the MS via the spray nozzle on the chip. NanoMate parameters were: spray voltage 1.7 kV and 0.6 psi N_2 _gas. The mass spectrometer was tuned on m/z 566.89. The full AGC target was set to 30000. The Orbitrap was externally calibrated in positive mode using sodium formate clusters in the range m/z 158.69-1110.79. For performing the MS^n ^experiments in MS1 a window of 1 m/z was used to isolate the mass of interest. The resolution used in the MS1 scan event was 60.000. For all dependent scan events (MSn) a resolution of 15000 and normalized collision energy 35% was used.

MS^n ^description:

MS 1 Full scan with limited m/z range, m/z of interest ± 0.5 m/z

MS 2 fragmentation of most intense m/z of MS1

MS 3 fragmentation of five most intense fragments in MS2

MS 4 fragmentation of five most intense fragments in MS3

MS 5 fragmentation of three most intense fragments in MS4

Following the sample infusion and MS analysis, the pipette tip was ejected and a new tip and nozzle were used for each sample, thereby preventing any cross-contamination or carry-over.

### Data analysis

#### NMR data analysis

##### Pre-processing of the data and alignment

A visual inspection of the technical replicates (4-fold) of each sample was carried out to ensure a high degree of reproducibility. The NMR data were pre-processed and aligned using a program developed in-house. This program was described in the Materials and Methods section of the paper by Lommen et al. [32], but has been adapted to run under Windows.

##### Normalization

The aligned fingerprint data in the form of generated spreadsheets were normalized using factors obtained from the scaling on the phospholipid signals of the ^1^H NMR spectra of the apolar fraction (see RESULTS section).

##### Statistical analysis

The normalized spreadsheets of both datasets were subjected to statistical analysis using Genemaths XT [79]. Standard initial analysis entailed performing a ^2^Log transformation and a PCA (average of rows and columns subtracted). This was followed by a ^2^Log transformation, a pre-selection of variables using an ANOVA (p < 0.01) followed by a PCA (average of rows and columns subtracted). An example of the effect of the ANOVA is given in Figure S2 in the Additional File 1. The grouping in the ANOVA was on the replicates per treatment for each passage number of cells when the effect of passage number as well as treatment was studied (10 groups with 5 replicates). The grouping in the ANOVA was on all replicates per treatment irrespective of passage number when only the effect of treatment was studied (2 groups of each 25 samples). From the latter figure a selection of peak loadings - underlying the separation by treatment in the PCA - could be exported as described in [31] (an example is given in Additional File 1, Figure S3); to check that the numbers of variables selected exceeded that expected purely by chance, table S1 was added in the Additional File 1. Only differences with a fold change higher than 1.2 and surviving a Bonferroni false positive correction were taken into account.

##### Identification of metabolites

The identification of relevant signals from the NMR data was carried out by using commercially available standards and/or from literature and databases, such as HMDB [80].

#### MS data analysis

##### Pre-processing of the data and alignment

A visual inspection of the technical replicates (4-fold) of each sample was carried out to ensure that high degree of reproducibility. The GC-MS and LC-MS data were pre-processed and aligned using MetAlign [31,34]. In short, this software performs a baseline correction, accurate mass calculation, data smoothing and noise reduction, followed by alignment between chromatograms, generating data files that are reduced in size 100-1000 times. The pre-processing and alignment parameters used have been included as Figures S4 and S5 in Additional File 1.

##### Normalization

#### LC-MS data

The aligned fingerprint data of the polar fraction dataset, in the form of generated spreadsheets, were normalized using the phospholipid scaling factors obtained from the ^1^H NMR spectra of the apolar fraction (see RESULTS section).

#### GC-MS data

The identified peaks of GC-MS chromatograms were manually integrated. This fraction was normalized by scaling to the raw values of the integrals of docosahexaenoic and arachidonic methyl esters (see RESULTS section).

##### Statistical analysis

The same procedure as described in the NMR section was used.

##### Identification of metabolites

A method of facilitating further analysis and identification was accomplished using GM2MS, an application of MetAlign that re-creates "new chromatograms" that contain only the peaks exported from the PCA selection [31]. Polar metabolites were identified with commercially available standards, with FT-MS/MS analysis and using databases, such as the HMDB [80]. Fatty acids were identified using the eluting order of the peaks and the NIST mass spectral library.

## Authors' contributions

AP, JK, J van D and DJ participated in the design of the study and overall discussion; CH helped to set up the methodology for *in vitro *metabolomics experiments; ARA optimized protocols, performed cell culture, exposures and collection of data; ARA and AL analysed data and interpreted the results. All authors read and approved the final manuscript. None of the authors had any personal or financial conflict of interest.

## Supplementary Material

Additional file 1 **PDF file containing Figures S1, S2, S3, S4 and S5 and Tables S1, S2, S3, S4, S5 and S6, mentioned in the text**. Click here for file

Additional file 2 **Metadata and NMR raw data: ZIP file containing**: -All NMR raw data (in JCAMP-DX format ".dx") -The metadata on NMR (polars & apolars), LC-MS and GC-MS data sets (as Tab delimited files ".txt"). Click here for file
