# Supplementary material for: An untargeted multi-technique metabolomics approach to studying intracellular metabolites of HepG2 cells exposed to 2,3,7,8-tetrachlorodibenzo-p-dioxin
Source: BMC Genomics. 2011 May 20;12:251. doi: 10.1186/1471-2164-12-251 (PMC3141663; doi:10.1186/1471-2164-12-251)
Supplement: Additional file 1 — PDF file containing Figures S1, S2, S3, S4 and S5 and Tables S1, S2, S3, S4, S5 and S6, mentioned in the text. [file 1471-2164-12-251-S1.PDF]

An untargeted multi-technique metabolomics approach to study intracellular metabolites of HepG2 cells exposed to 2,3,7,8-tetrachlorodibenzo-p-dioxin

Ainhoa Ruiz Aracama[1, 3*], *Ad Peijnenburg*[1, 3], *Jos Kleinjans*[2, 3], *Danyel Jennen*[2, 3], *Joost van Delft*[2, 3], *Caroline Hellfrisch*[1], *Arjen Lommen*[1, 3]

[1] RIKILT-Institute of Food Safety, Wageningen University and Research Centre, P.O. Box 230, 6700 AE, Wageningen, The Netherlands

[2] Department of Health Risk Analysis and Toxicology, P.O. Box 616, 6200 MD Maastricht University, Maastricht ,The Netherlands

[3] Netherlands Toxicogenomics Centre, The Netherlands

[*]Corresponding author: Email: Ainhoa.ruiz@wur.nl

ADDITIONAL FILE 1

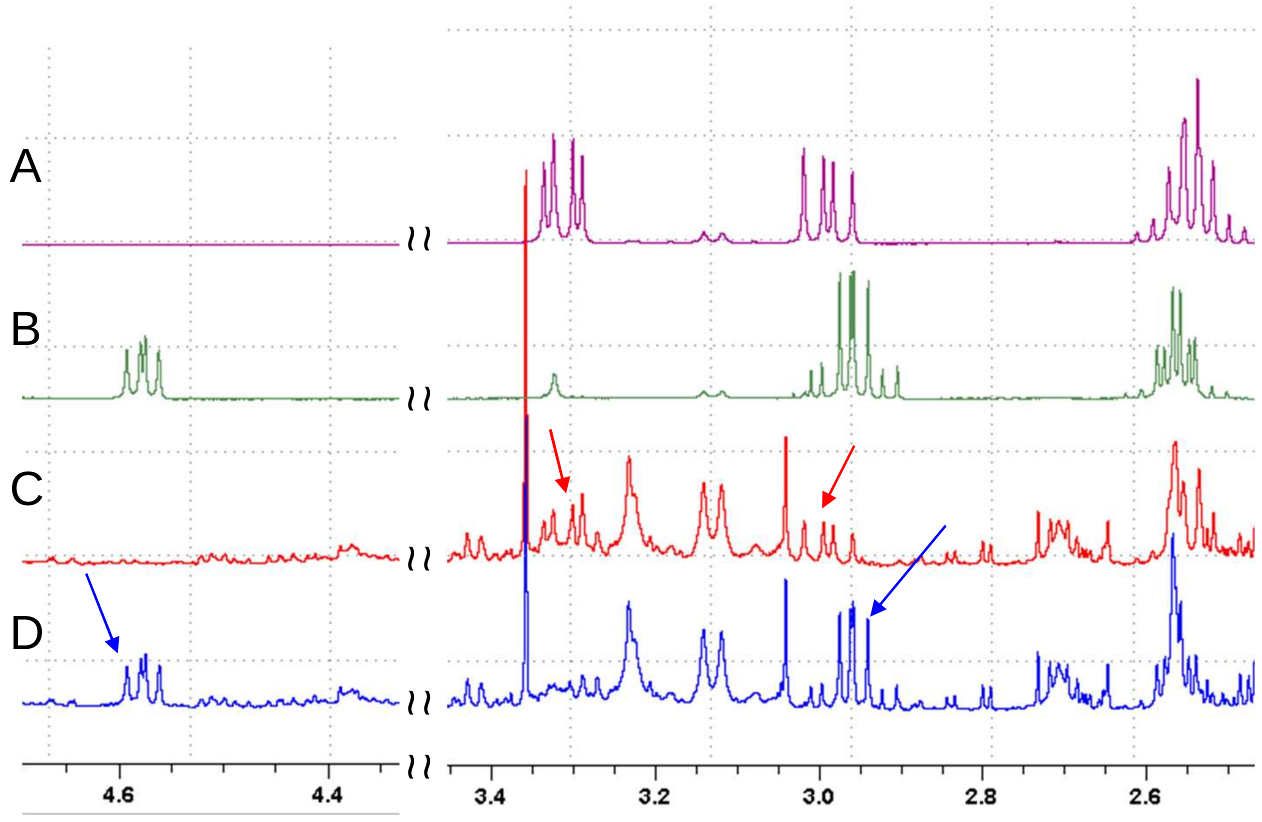

Figure S1. Enlarged ^{1}H NMR spectral regions showing the characteristic GSH and GSSG signals. **D:** Polar metabolites of HepG2 cells analyzed at pH=7 directly after extraction; **C:** Same extract of polar metabolites of HepG2 cells analyzed after 9 months of storage at -20°C, pH=7; **B:** GSH spectrum; **A:** GSSG spectrum

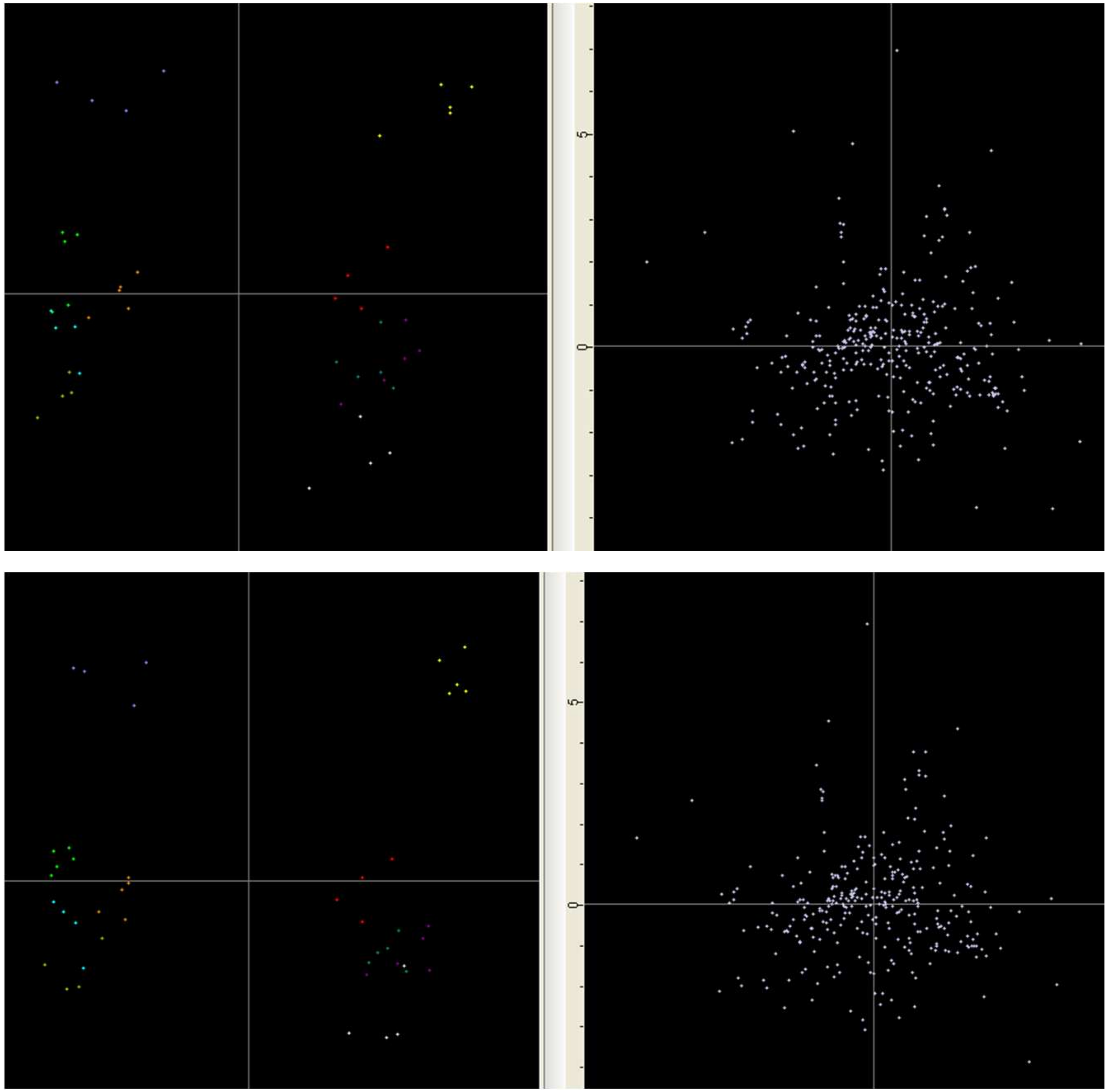

Figure S2. Example of PCA plots A) before ANOVA; B) after ANOVA p<0.01. On the left, spheres with the same color are technical replicates of the same sample per passage number. On the right, the PCA loadings.

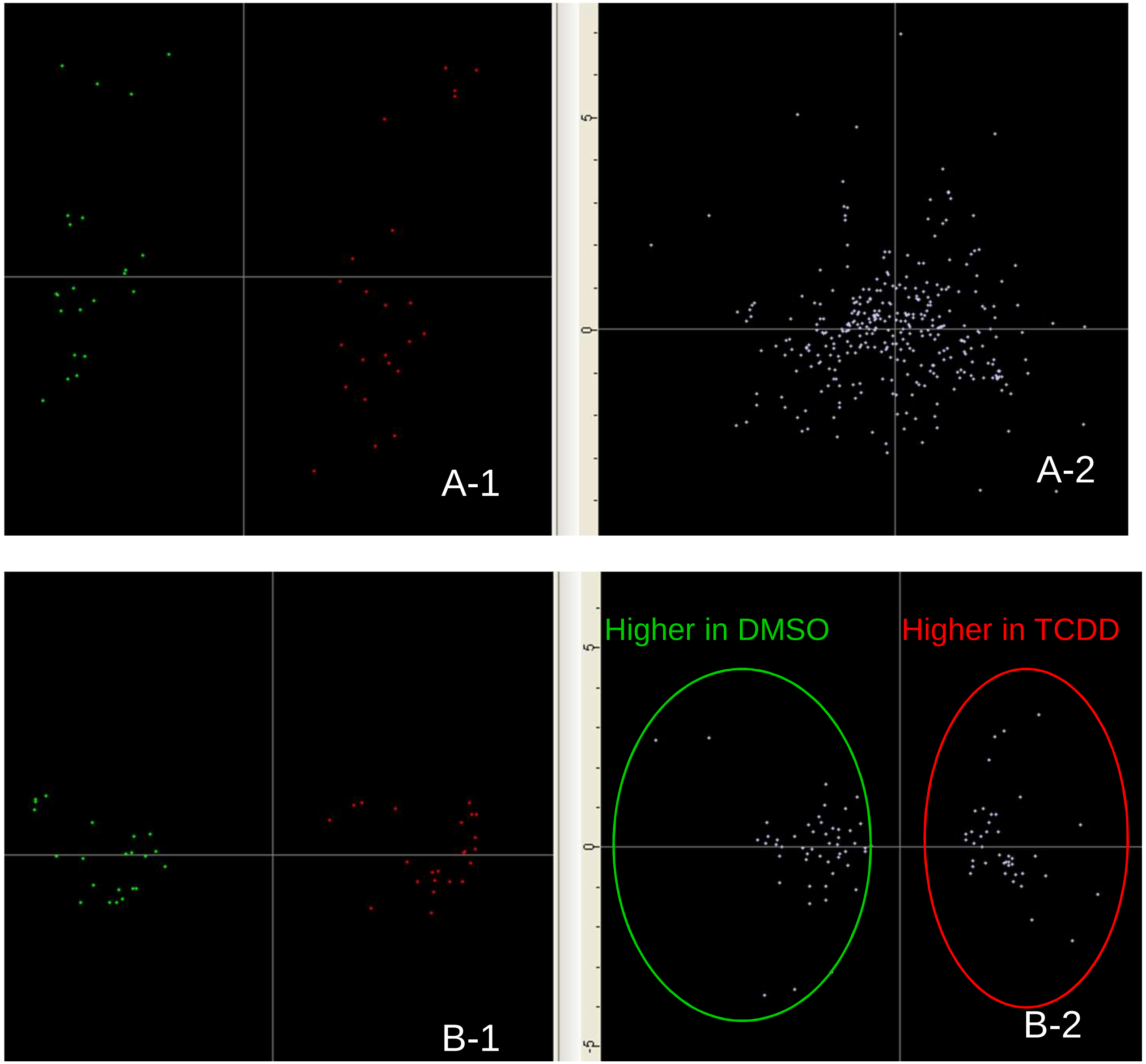

Figure S3. Example of PCA plots A) before ANOVA; B) After ANOVA $p<0.01$. On the left, samples coloured with regard to the treatment (DMSO: green; TCDD: red). On the right (B-2), PCA loadings of the peaks significantly affected by the treatment and used for further identification of the effects of TCDD .

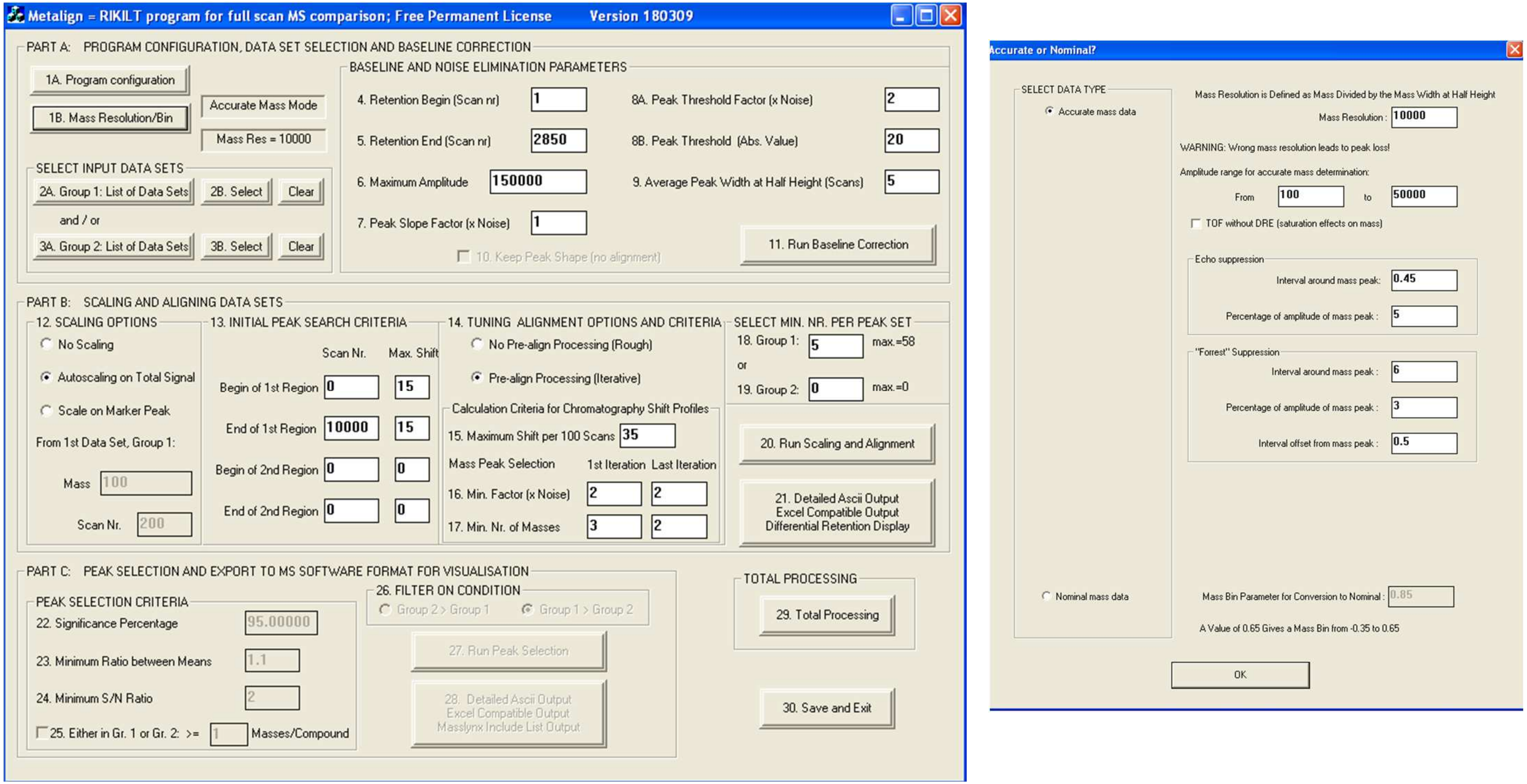

Figure S4: MetAlign parameters used for LC-MS data processing and alignment

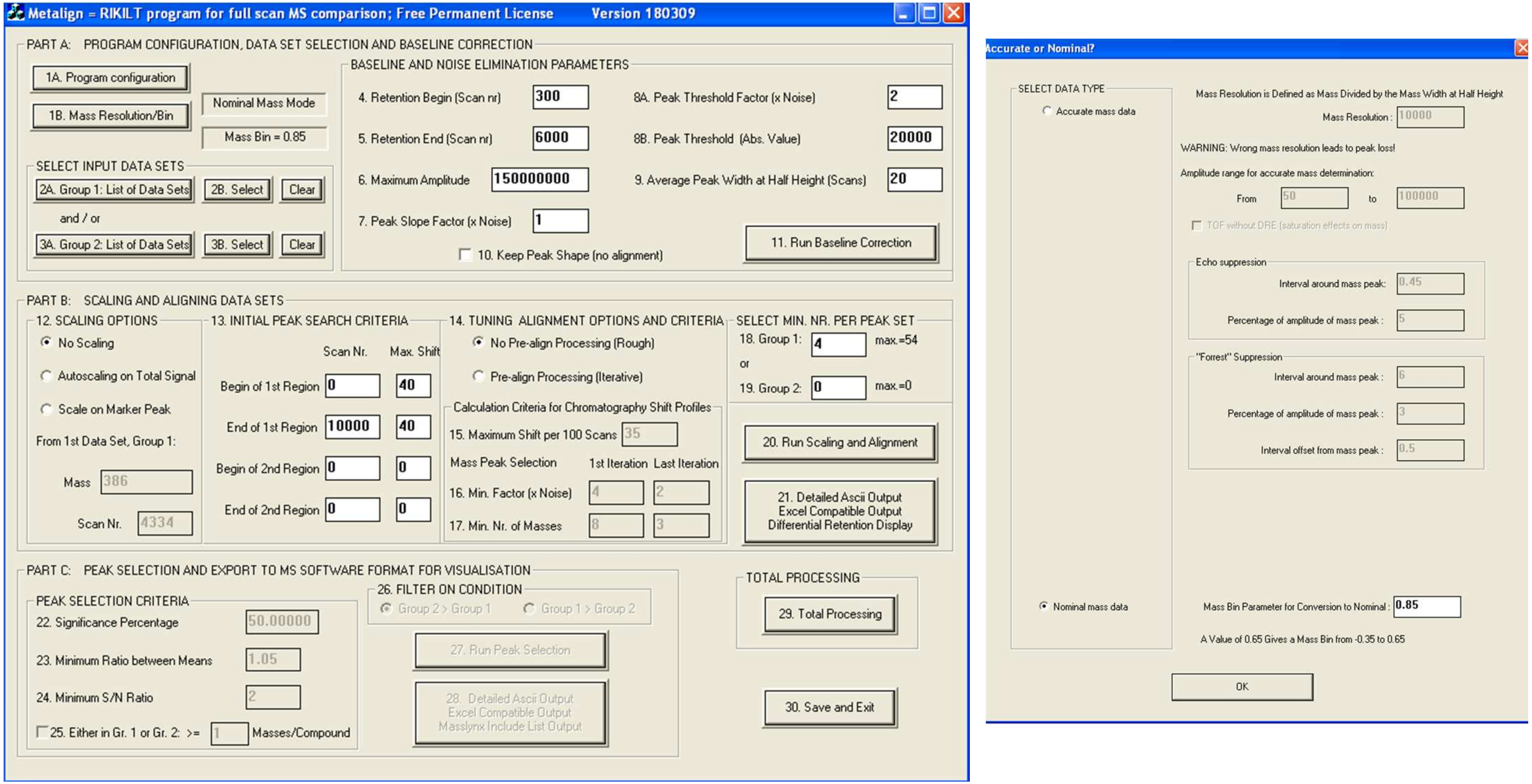

Figure S5: MetAlign parameters used for GC-MS data processing and alignment

Table S1. Numbers of surviving peaks after statistical selection when grouping all samples with regard to the treatment (2 groups –DMSO *vs* TCDD-; 25 samples in each)

	**ANOVA p<0.01**	**ANOVA p<0.01 Bonferroni**
**Apolars NMR**	39% (66/170)	24% (40/170)
**Apolars GC-MS**	33% (262/793)	19% (151/793)
**Polars NMR**	53% (178/332)	23% (75/332)
**Polars LC-MS**	30% (310/1037)	12% (124/1037)

Effect of TCDD on cell number estimated from NMR spectra and by cell counting.

Tables S2-1 & 2 are from different experiments because in practice it is not possible to do harvesting and counting on the same flasks.

Table S2-1. PL ratios calculated from the NMR (determined from 4 independent flasks).

	**D-1**	**D-2**	**D-3**	**D-4**	**$D_{average}$**	**D_{stdv}**	**T-1**	**T-2**	**T-3**	**T-4**	**$T_{average}$**	**Ts_{tdv}**
***PL/$CHCl_3$**	64	84	77	73	**74.5**	**8.3**	53	56	35	50	**48.5**	**9.3**

*values obtained multiplying the ratio PL/$CHCl_3$ by 100000

Table S2-2. Number of cells counted on of 3 different flasks using the Neubauer chamber after trypsinization.

	**D-1**	**D-2**	**D-3**	**$D_{average}$**	**D_{stdv}**	**T-1**	**T-2**	**T-3**	**$T_{average}$**	**Ts_{tdv}**
**Cell number**	131	118	88	**112.3**	**22.1**	66	61	71	**66.0**	**5.0**

Table S3. Fold change value for ^{1}H NMR proton signals of apolar metabolites significantly affected by TCDD (selected from MVA after ANOVA, p<0.01), for each passage number and for the average.

	**Proton assignment**	**p7**	**p11**	**p11n**	**p17**	**p30**	**Average each**
Olefines	-C**H**=C**H**-	**0.58** (0.03)	**0.75** (0.12)	**0.76** (0.05)	**0.75** (0.11)	**0.87** (0.20)	**0.74** (0.27)
Glycerol	-C$\mathbf{H_2}$OCOR >C**H**OCOR	**0.24** (0.19)	**0.42** (0.20)	**0.57** (0.14)	**0.59** (0.16)	**0.74** (0.13)	**0.51** (0.37)
Bis-allylic	=HC-C$\mathbf{H_2}$-CH=	**0.81** (0.04)	**0.89** (0.06)	**0.90** (0.05)	**0.93** (0.09)	**0.95** (0.14)	**0.90** (0.19)
Allylic	-C$\mathbf{H_2}$-CH=CH-	**0.63** (0.03)	**0.78** (0.10)	**0.75** (0.04)	**0.76** (0.09)	**0.88** (0.14)	**0.76** (0.20)
Cholesterol ester	$C^{19}\mathbf{H_3}$	**0.52** (0.05)	**0.73** (0.16)	**0.63** (0.08)	**0.73** (0.10)	**0.83** (0.24)	**0.69** (0.32)

Table S4. Fold change value for fatty acids (determined by GC-MS) significantly affected by TCDD (selected from MVA after ANOVA, p<0.01), for each passage number and for the average.

	p7	p11	p11n	p17	p30	Average
**C12:0**	**0.63**	**0.74**	**0.73**	**0.85**	**0.93**	**0.78**
	(0.12)	(0.09)	(0.12)	(0.09)	(0.13)	(0.25)
**C14:0**	**0.60**	**0.70**	**0.68**	**0.76**	**0.84**	**0.72**
	(0.16)	(0.14)	(0.11)	(0.04)	(0.12)	(0.30)
**C14:1a**	**0.54**	**0.69**	**0.67**	**0.80**	**0.78**	**0.70**
	(0.21)	(0.12)	(0.22)	(0.07)	(0.16)	(0.19)
**C14:1b**	**0.16**	**0.31**	**0.28**	**0.31**	**0.38**	**0.29**
	(0.28)	(0.08)	(0.10)	(0.08)	(0.11)	(0.26)
**C16:1n6**	**0.49**	**0.68**	**0.65**	**0.65**	**0.70**	**0.63**
	(0.09)	(0.10)	(0.09)	(0.03)	(0.09)	(0.20)
**C16:1n9**	**0.52**	**0.64**	**0.65**	**0.63**	**0.66**	**0.62**
	(0.22)	(0.11)	(0.10)	(0.06)	(0.12)	(0.22)
**C17:0**	**1.39**	**1.13**	**1.21**	**1.04**	**1.23**	**1.20**
	(0.19)	(0.04)	(0.05)	(0.02)	(0.10)	(0.21)
**C18:0**	**1.37**	**1.25**	**1.23**	**1.19**	**1.32**	**1.27**
	(0.13)	(0.15)	(0.11)	(0.07)	(0.15)	(0.19)
**C18:1a**	**0.67**	**0.81**	**0.77**	**0.84**	**0.83**	**0.78**
	(0.18)	(0.10)	(0.11)	(0.02)	(0.10)	(0.23)
**C18:1c**	**0.60**	**0.77**	**0.72**	**0.73**	**0.79**	**0.72**
	(0.12)	(0.11)	(0.05)	(0.10)	(0.07)	(0.22)
**C18:2a**	**0.62**	**0.72**	**0.79**	**0.74**	**0.68**	**0.71**
	(0.09)	(0.08)	(0.09)	(0.08)	(0.07)	(0.18)
**C18:2b**	**0.39**	**0.72**	**0.62**	**0.70**	**0.72**	**0.63**
	(0.20)	(0.22)	(0.26)	(0.11)	(0.17)	(0.33)
**C18:2c**	**1.42**	**1.25**	**1.26**	**1.24**	**1.28**	**1.29**
	(0.19)	(0.15)	(0.09)	(0.10)	(0.18)	(0.26)
**C18:2d**	**0.56**	**0.64**	**0.67**	**0.66**	**0.68**	**0.64**
	(0.25)	(0.06)	(0.09)	(0.12)	(0.28)	(0.27)
**C18:2e**	**0.53**	**0.65**	**0.62**	**0.75**	**0.59**	**0.63**
	(0.16)	(0.27)	(0.29)	(0.18)	(0.07)	(0.35)
**C20:1b**	**0.74**	**0.77**	**0.81**	**0.82**	**0.80**	**0.79**
	(0.13)	(0.02)	(0.05)	(0.03)	(0.05)	(0.16)
**C20:2a**	**0.66**	**0.79**	**0.73**	**0.77**	**0.75**	**0.74**
	(0.12)	(0.04)	(0.07)	(0.02)	(0.06)	(0.14)
**C20:2b**	**0.63**	**0.73**	**0.72**	**0.74**	**0.68**	**0.70**
	(0.09)	(0.03)	(0.06)	(0.04)	(0.05)	(0.20)
**C20:3a**	**0.77**	**0.82**	**0.80**	**0.82**	**0.76**	**0.80**
	(0.06)	(0.03)	(0.03)	(0.03)	(0.02)	(0.12)
**C20:3b**	**0.60**	**0.62**	**0.63**	**0.64**	**0.70**	**0.64**
	(0.10)	(0.10)	(0.07)	(0.07)	(0.07)	(0.23)
**C22:2**	**0.61**	**0.61**	**0.58**	**0.68**	**0.59**	**0.61**
	(0.27)	(0.22)	(0.21)	(0.04)	(0.12)	(0.33)
**C22:3a**	**0.70**	**0.74**	**0.70**	**0.74**	**0.80**	**0.73**
	(0.09)	(0.03)	(0.03)	(0.02)	(0.04)	(0.09)

Table S5. Fold change value for ^{1}H NMR proton signals of polar metabolites significantly affected by TCDD (selected from MVA after ANOVA, $p<0.01$), for each passage number and for the average.

	p7	p11	p11n	p17	p30	Average
**Leucine. Isoleucine**	**0.62**	**0.71**	**0.68**	**0.68**	**0.74**	**0.69**
	(0.08)	(0.06)	(0.13)	(0.13)	(0.05)	(0.27)
**Valine**	**0.61**	**0.75**	**0.73**	**0.71**	**0.80**	**0.72**
	(0.20)	(0.12)	(0.14)	(0.14)	(0.17)	(0.33)
**Alanine**	**0.62**	**0.69**	**0.68**	**0.67**	**0.72**	**0.68**
	(0.13)	(0.07)	(0.15)	(0.15)	(0.09)	(0.15)
**N-acetyl aspartate**	**0.56**	**0.75**	**0.71**	**0.62**	**0.75**	**0.68**
	(0.07)	(0.09)	(0.14)	(0.14)	(0.05)	(0.32)
**Glutamate**	**0.76**	**0.78**	**0.82**	**0.76**	**0.83**	**0.79**
	(0.09)	(0.08)	(0.11)	(0.11)	(0.06)	(0.26)
**Glutamine**	**0.57**	**0.73**	**0.68**	**0.71**	**0.76**	**0.69**
	(0.07)	(0.07)	(0.15)	(0.15)	(0.07)	(0.20)
**Glycine**	**0.63**	**0.73**	**0.71**	**0.72**	**0.81**	**0.72**
	(0.09)	(0.05)	(0.12)	(0.12)	(0.08)	(0.23)
**Aspartate**	**0.5**	**0.72**	**0.64**	**0.68**	**0.76**	**0.66**
	(0.04)	(0.06)	(0.10)	(0.10)	(0.10)	(0.29)
**Serine**	**0.62**	**0.69**	**0.6**	**0.61**	**0.74**	**0.65**
	(0.07)	(0.07)	(0.09)	(0.09)	(0.07)	(0.28)
**Tyrosine**	**0.48**	**0.59**	**0.58**	**0.56**	**0.68**	**0.58**
	(0.07)	(0.09)	(0.10)	(0.10)	(0.07)	(0.31)
**Lactate**	**0.39**	**0.48**	**0.53**	**0.47**	**0.58**	**0.49**
	(0.05)	(0.03)	(0.10)	(0.10)	(0.06)	(0.21)
**Creatine/phosphocreatine**	**0.63**	**0.68**	**0.64**	**0.61**	**0.75**	**0.66**
	(0.06)	(0.06)	(0.10)	(0.10)	(0.07)	(0.28)
**Oxidized glutathione**	**1.09**	**1.15**	**1.33**	**1.29**	**1.44**	**1.26**
	(0.07)	(0.09)	(0.11)	(0.15)	(0.10)	(0.32)
**Reduced glutathione**	**1.88**	**1.73**	**1.35**	**1.69**	**1.27**	**1.58**
	(0.07)	(0.10)	(0.08)	(0.12)	(0.06)	(0.29)
**Taurine**	**1.45**	**1.38**	**1.4**	**1.43**	**1.21**	**1.37**
	(0.08)	(0.08)	(0.10)	(0.12)	(0.07)	(0.22)
**Citrate**	**1.52**	**1.48**	**1.45**	**1.45**	**1.38**	**1.46**
	(0.09)	(0.10)	(0.10)	(0.12)	(0.06)	(0.26)

Table S6. Fold change value for polar metabolites (determined by LC-MS) significantly affected by TCDD (selected from MVA after ANOVA, p<0.01), for each passage number and for the average.

	p7	p11	p11n	p17	p30	Average
**Leucine/Isoleucine**	**0.51**	**0.63**	**0.69**	**0.67**	**0.70**	**0.64**
	(0.10)	(0.09)	(0.11)	(0.13)	(0.12)	(0.25)
**Valine**	**0.67**	**0.61**	**0.71**	**0.68**	**0.76**	**0.69**
	(0.09)	(0.16)	(0.14)	(0.12)	(0.08)	(0.27)
**N-acetyl-aspartate**	**0.73**	**0.70**	**0.67**	**0.65**	**0.84**	**0.72**
	(0.11)	(0.18)	(0.09)	(0.12)	(0.19)	(0.31)
**Glutamate**	**0.74**	**0.87**	**0.71**	**0.91**	**0.78**	**0.80**
	(0.10)	(0.13)	(0.10)	(0.22)	(0.14)	(0.32)
**Tyrosine**	**0.36**	**0.44**	**0.48**	**0.44**	**0.50**	**0.44**
	(0.15)	(0.10)	(0.07)	(0.14)	(0.11)	(0.26)
**Proline**	**0.52**	**0.50**	**0.53**	**0.50**	**0.67**	**0.54**
	(0.07)	(0.08)	(0.12)	(0.10)	(0.11)	(0.15)
**Tryptophan**	**0.75**	**0.71**	**0.80**	**0.83**	**0.86**	**0.79**
	(0.05)	(0.08)	(0.09)	(0.13)	(0.15)	(0.18)
**Spermidine**	**0.22**	**0.28**	**0.36**	**0.24**	**0.11**	**0.24**
	(0.06)	(0.08)	(0.08)	(0.21)	(0.28)	(0.37)
**N-acetyl-spermidine**	**0.53**	**0.64**	**0.61**	**0.68**	**0.49**	**0.59**
	(0.11)	(0.12)	(0.05)	(0.14)	(0.08)	(0.23)
**Panthotenic acid**	**0.51**	**0.64**	**0.62**	**0.65**	**0.69**	**0.62**
	(0.06)	(0.09)	(0.15)	(0.19)	(0.14)	(0.30)
**Propionylcarnitine**	**0.62**	**0.70**	**0.71**	**0.68**	**0.58**	**0.66**
	(0.12)	(0.14)	(0.12)	(0.13)	(0.16)	(0.30)
**Butyrylcarnitine**	**0.51**	**0.65**	**0.68**	**0.61**	**0.47**	**0.58**
	(0.08)	(0.17)	(0.13)	(0.13)	(0.16)	(0.30)
**UMP**	**0.60**	**0.65**	**0.79**	**0.73**	**0.77**	**0.71**
	(0.27)	(0.15)	(0.16)	(0.12)	(0.10)	(0.38)
**AMP**	**0.84**	**0.83**	**0.62**	**0.61**	**0.47**	**0.67**
	(0.14)	(0.23)	(0.13)	(0.15)	(0.24)	(0.41)
**Reduced glutathione**	**1.47**	**1.21**	**1.13**	**1.25**	**1.08**	**1.23**
	(0.12)	(0.18)	(0.07)	(0.20)	(0.13)	(0.36)
**Oxidized glutathione**	**1.14**	**1.15**	**1.38**	**1.41**	**1.44**	**1.31**
	(0.06)	(0.09)	(0.11)	(0.13)	(0.15)	(0.28)
**Citri/Isocitric acid**	**1.56**	**1.45**	**2.02**	**1.46**	**1.80**	**1.68**
	(0.17)	(0.12)	(0.20)	(0.17)	(0.18)	(0.38)
**UMP-N-acetyl glucosamine/galactosamine**	**2.04**	**1.16**	**1.23**	**1.12**	**1.08**	**1.48**
	(0.15)	(0.22)	(0.18)	(0.21)	(0.17)	(0.43)
